# Misfit of Implant-Supported Zirconia (Y-TZP) CAD-CAM Framework Compared to Non-Zirconia Frameworks: A Systematic Review

**DOI:** 10.3390/medicina58101347

**Published:** 2022-09-25

**Authors:** Hussain D. Alsayed

**Affiliations:** Prosthetic Dental Science Department, College of Dentistry, King Saud University, Riyadh 60169, Saudi Arabia; halsayed@ksu.edu.sa; Fax: +966-1467-8639

**Keywords:** systematic review, misfit, implant frameworks, Zirconium, metal alloys

## Abstract

*Objective:* The aim of the study was to systematically review the overall outcomes of studies comparing the misfit of yttria-stabilized zirconia (Y-TZP) CAD-CAM implant-supported frameworks with frameworks fabricated with other materials and techniques. *Methods:* An electronic literature search of English literature was performed using Google Scholar, Scopus, Web of Science, MEDLINE (OVID), EMBASE, and PubMed, using predetermined inclusion criteria. Specific terms were utilized in conducting a search from the inception of the respective database up to May 2022. After the search strategy was applied, the data were extracted and the results were analyzed. The focused question was: Is the misfit of the implant-supported zirconia CAD-CAM framework lower than that of non-Y-TZP implant-supported fixed restorations? *Results:* Eleven articles were included for qualitative assessment and critical appraisal in this review. In the included studies, Y-TZP CAD-CAM implant-supported frameworks were compared to Titanium (Ti), Ni-Cr, Co-Cr, PEEK and high-density polymer, and cast and CAD-CAM frameworks. The studies used scanning electron microscopy, one-screw tests, digital or optical microscopy, 3D virtual assessment, and replica techniques for analyzing the misfit of frameworks. Six studies showed comparable misfits among the Y-TZP CAD-CAM frameworks and the controls. Three studies showed higher misfits for the Y-TZP CAD-CAM frameworks, whereas two studies reported lower misfits for Y-TZP CAD-CAM implant frameworks compared to controls. *Conclusion:* Y-TZP CAD-CAM implant-supported frameworks have comparable misfits to other implant-supported frameworks. However, due to heterogeneity in the methodologies of the included studies, the overall numerical misfit of the frameworks assessed in the reviewed studies is debatable

## 1. Introduction

Dental implants are surgically placed devices that have direct contact with the alveolar bone [1,2]. In addition to supporting single-tooth restorations, they are also used to support and retain prostheses for the restoration of partially or completely edentulous patients [3]. Implant-supported removable and fixed prostheses possess significant advantages over conventional prostheses. In addition to offering superior support [4] and stability [5], implant-supported prostheses preserve residual bone [6] and are esthetically pleasing [7]. It has been estimated that the 5-year success-rate of implant-supported prostheses is as high as 95% [8,9].

Frameworks of implant-supported dentures have conventionally been constructed from cast metals [10]. However, cast implant-supported prostheses have several drawbacks. The clinical phase of these prostheses includes taking impressions which may become easily distorted and damaged during or after the impression-taking process [11]. In addition, the cast metal alloys may undergo distortion during the casting process, resulting in a misfit of up to 450 µm [12,13]. Moreover, the wax pattern of the cast framework may also undergo dimensional changes, resulting in a misfit of the prosthesis [14]. Ideally, a framework should fit passively by not exerting biologically detrimental forces on the supportive teeth, the supportive tissues, and the framework [15]. Furthermore, there should be no gap between the margins of the framework and the supportive tissues and teeth. The misfit is measured by evaluating the distance between the final restoration and the corresponding fitting surfaces. Although the misfit of cast prostheses may be reduced by sectioning and then re-connecting the framework, the mechanical properties of the cast metal may be diminished, which can lead to fractures of the prostheses [16]. Additionally, misfit causes micro-gaps between the implant and the framework. This gap harbors bacteria which may cause infection of the peri-implant tissues [17]. A misfitting framework can also lead to the loosening or even the fracture of prosthetic implant screws [18]. Eventually, long-standing misfit results in the instability of the framework, inducing failure of the dental implants [19].

Over the last few years, prostheses designed and constructed via computer-aided design and computer-aided manufacturing (CAD-CAM) have gained popularity [20]. Briefly, the CAD-CAM process involves three-dimensional (3D) digital scanning of the teeth and related structures in the oral cavity to produce a virtual 3D model. The virtual model is then processed by a computer connected to a milling machine that constructs the prostheses. The milling system produces a prosthesis from a block of homogenous material such as Titanium (Ti) or yttria-stabilized zirconia (Y-TZP) [21]. Studies have indicated that CAD-CAM-constructed prostheses have a significantly lower misfit compared to cast frameworks [22]. There are two types of CAD-CAM systems: additive and subtractive [20]. Additive manufacturing focuses on building appliances and objects layer by layer, while subtractive systems remove material from pre-formed blocks into appliances. Subtractive manufacturing has seen more clinical use than additive manufacturing; however, the latter has gained popularity in the last few years [20]. A recent systematic review of in vitro and clinical studies indicated that CAD-CAM frameworks have significantly better fits compared to cast frameworks [23].

Y-TZP has been a popular material for the construction of CAD-CAM implant-supported frameworks over the last decade, and its market-share is expected to double by 2024 [24]. Indeed, Y-TZP frameworks exhibit exceptional strength and fracture toughness [25]. Clinical studies suggest that Y-TZP frameworks remain stable for more than 5 years post-insertion [26]. Moreover, due to its higher color stability and the biocompatibility and accuracy of CAD-CAM fabrication, Y-TZP presents an attractive alternative to metal alloys from the patients’ perspective [27]. Several in vitro studies have compared the fit (or misfit) of metal alloys and Ti and polymer frameworks with that of Y-TZP CAD-CAM frameworks [28,29,30,31,32,33,34,35,36,37,38]. In a study by Abduo et al., the vertical misfits for Y-TZP and Ti CAD-CAM frameworks were comparable [28]. By contrast, in a study by de Rio Silva et al., Ti CAD-CAM frameworks had a lower misfit compared to Y-TZP frameworks [38]. A controversy exists among the studies reporting the misfit of Y-TZP CAD-CAM with other materials and techniques. So, the aim was to systematically review the overall outcomes of studies comparing the misfit of Y-TZP CAD-CAM implant-supported frameworks with frameworks fabricated with other materials and techniques. I hypothesize that, overall, the misfit of Y-TZP CAD-CAM frameworks will be lower compared to that of frameworks fabricated with other materials.

## 2. Materials and Methods

### 2.1. Focused Question

Following the Participants, Intervention, Control, and Outcomes principal described in the Preferred Reporting Items in Systematic Reviews and Meta-Analysis (PRISMA) statement [39], the following focused question was constructed: ‘Is the misfit of implant-supported Zirconia CAD-CAM frameworks lower than that of non-Y-TZP implant-supported fixed restorations?’ (Participants: Patients or study casts; Intervention: Y-TZP CAD-CAM implant-supported dental prostheses; Controls: Non-Y-TZP-supported fixed restorations; Outcomes: Misfit).

### 2.2. Eligibility Criteria

Before conducting the literature search, eligibility criteria were decided on by the author. Prospective clinical studies, case reports and series, animal studies, and laboratory studies focusing on comparing the fit or misfit of CAD-CAM implant-supported Zirconia fixed restorations with other non-Y-TZP implant-supported restorations were included. Literature from inception to May 2022 was searched. Additionally, only articles in English were included. Studies not in the English language, systematic or literature reviews, and letters to the editor were excluded.

### 2.3. Literature Search

An electronic search using the keywords ((Zirconia) OR (Y-TZP) AND (Restoration or bridge or framework) AND ((computer-aided design OR CAD)) or (computer-aided manufacture) OR CAM)) AND (full arch OR partial OR complete) AND (control OR titanium OR resin OR cobalt chromium) AND (misfit OR gap OR adaptation) AND (implant)) was conducted on the following databases: PubMED/MEDLINE, ISI Web of Science/Knowledge, Scopus, Embase, and Google Scholar, including studies up to May 2022. Following the exclusion of the non-relevant articles on the basis of titles and abstracts, the full texts of studies appearing to meet the inclusion criteria were downloaded. Additionally, the reference lists of the full-text documents were scanned manually to look for relevant articles. Furthermore, a similar search was repeated using the same keywords on the clinical trial registers CONTROL and clinicaltrials.gov. The literature search was conducted by author (HA) interpedently, and any disagreements were solved by discussion with a statistician. 

### 2.4. Data Extraction

Using predetermined items, the data from each study were extracted to construct tables. Briefly, the materials used to construct the dentures in the test and control groups (if any), the method of denture fabrication, the type of misfit (or fit) assessment employed, measurements of any other variables, and the qualitative outcomes of the studies were summarized in the first table. Summarized information on the implant or abutment system, the dimensions and positions of the dental implants, the type of implant-supported prostheses (fixed or removable, along with the number of units), the CAD-CAM fabrication system, and the numerical values of the misfit or fit was also prepared.

### 2.5. Quality Assessment

The overall quality of the studies and any bias present in the studies were assessed using a modified version of the ‘Guidelines For Reporting Pre-Clinical In Vitro Studies On Dental Materials’ developed by Mariano [40]. Briefly, in each study, the following items were assessed: an adequate abstract, introduction (background and objects), and methodology (replicability, reporting of adequate outcomes, a predetermined sample size, and details of any randomization, blinding, or concealment employed), adequate statistics, a mention of any limitations in the discussion, funding details, and, if any, the protocol of the study was accessible. A 15-point checklist was used to grade each study. Each study was assigned an overall quality of low (score: 0–5), medium (score: 6–10), or high (score: 11–15). 

## 3. Results

### 3.1. Results of the Literature Search

**Figure 1:**
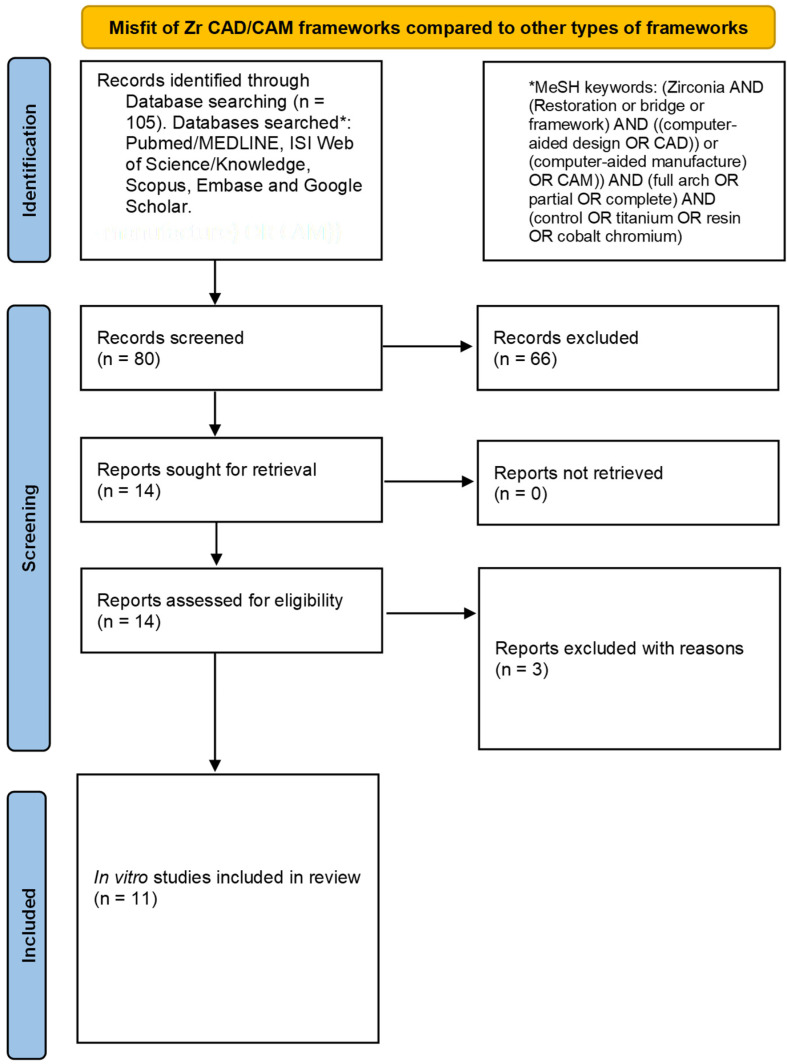
PRISMA flow diagram employed for the literature search.

The primary literature search resulted in 105 articles. 25 articles were eliminated on the basis of titles. Of the 80 articles, 66 articles were further excluded after the review of the abstracts and on the basis of relevance. Therefore, the full texts of 14 articles were downloaded to assess their eligibility for inclusion in this review. Three full-text articles were excluded because two of them were systematic reviews [41,42] and one did not include any controls to which to compare the misfit of the Y-TZP prosthesis [43]. Hence, 11 articles were included for qualitative assessment and critical appraisal in this review [28,29,30,31,32,33,34,35,36,37,38]. The study methodology is presented in Figure 1. The overall Kappa (intra-examiner reliability) score was calculated as 0.87.

### 3.2. General Characteristics

All studies included in this review were in vitro laboratory studies that compared the fit or misfit of Y-TZP CAD-CAM implant-supported frameworks with other materials or fabrication methods [28,29,30,31,32,33,34,35,36,37,38] (Table 1). In six studies, Ti CAD-CAM frameworks were included in the comparison groups [28,31,36,37,38]. In one study, cast Ni-Cr frameworks were included as a comparison [29], and Cast Co-Cr frameworks were compared with CAD-CAM Y-TZP in four studies [30,31,32,38]. In two studies, CAD-CAM was also used to construct Co-Cr frameworks as comparison groups [30,32], and in one study, mechanically scanned CAD-CAM Y-TZP frameworks were also tested [31]. CAD-CAM Y-TZP frameworks were compared with frameworks constructed from CAD-CAM polyetheretherketone (PEEK) and CAD-CAM resin composites in one study [33]. In one study, the effect of porcelain veneering on the misfit of Y-TZP and Ti CAD-CAM frameworks was assessed [34], and in another study, a CAD-CAM high-density polymer (HDP) framework was tested against CAD-CAM Y-TZP [35]. Copy-milled Y-TZP frameworks were constructed in three studies [29,38]. In addition to marginal or vertical misfit, four studies also compared cyclic fatigue [29], retention [33], loosening torque [37,38], and stress [38] between different frameworks.

In six studies, vertical misfit or fit was analyzed [28,29,30,31,32,35,36,37,38]. In one study, the internal misfit was assessed [33], and in another study, the three-dimensional (3D) misfit of the frameworks was assessed [34]. In four studies, scanning electron microscopy (SEM) was used to analyze the misfit [30,31,32,37]. In four studies, the one-screw test was used to analyze the misfit [31,35,36,38], and in two studies, digital or optical microscopy was used for fit analysis [28,29]. 3D virtual assessment was used to determine the misfit in one study [34], and the ‘replica technique’ was used to determine the internal misfit in one study [33].

In the studies reviewed, the following implant systems or brands were used: Nobel Biocare Active RP (three studies [30,34,35]), Mk III TiUnite by Nobel Biocare [28], Friatz by Dentsply [29], Replace Select^TM^ Tapered RP by Nobel Biocare [31], Titamax Cortical Ti by Neodent [32], an unspecified brand by Nobel Biocare [36], ITI Straumann [37], and Easy Grip Porous EH [38]. In one study, the implant system was not specified [33]. The length of the implants ranged from 9 mm to 13 mm and from 3.75 mm to 4.3 mm [28,29,30,31,32,33,34,35,36,37,38] (Table 2).

In three studies, three-unit fixed partial dentures (FDP) were constructed [29,30,32], and in five studies, full arch fixed dentures on four implants (all-on-four) were constructed [28,34,35,36,37]. In one study, a ten-unit fixed prosthesis supported by six implants was constructed [31], and in one study, six implants supported a fixed prosthesis [38]. In the included studies, the following CAD systems were used: Zirkozahn (four studies [34,35,36,37]), Nobel Biocare [28,31], Cerec 3 [29], 3Shape [33], and Ceramill Map [38]. The CAM systems were: M1 Milling Unit (three studies [34,35,36]), M5 Milling Unit [37], 3Shape [33], and Ceramill Motion [38]. In two studies, the CAD-CAM system was not specified [30,32].

### 3.3. Outcomes of Included Studies

In five studies, the misfits of the Y-TZP CAD-CAM frameworks were comparable to that of Ti CAD-CAM [28,31,35,36,37]. In one study, Ti CAD-CAM had a significantly lower misfit compared to Y-TZP CAD-CAM [38]. Compared to Co-Cr CAD-CAM, in one study, Y-TZP CAD-CAM exhibited a comparable fit [30], and in another one, Co-Cr CAD-CAM had a significantly better fit [32]. When compared to copy-milled Y-TZP and Ni-Cr CAD-CAM frameworks, Y-TZP CAD-CAM had a lower misfit in one study [29]. When compared with PEEK and resin composites, Y-TZP CAD-CAM prosthesis had a better fit [33]. On the other hand, in one study, CAD-CAM frameworks constructed from high-density polymer (HDP) had lower misfits than Y-TZP CAD-CAM frameworks [35].

### 3.4. Results of the Quality Assessment

Eight studies received an overall quality grade of ‘Medium’ [28,29,30,32,34,35,36,37], one study was graded as ‘Low’ [33], and only two studies were graded as ‘High’ [31,38] (Table 3). All studies contained an adequate abstract and described the statistical tests conducted [28,29,30,31,32,33,34,35,36,37,38]. All but one study contained an adequate introduction [28,29,30,31,32,34,35,36,37,38]. Although all studies contained an introduction [28,29,30,31,32,33,34,35,36,37,38], in one study, the objectives and the background were not adequately stated [33]. One study did not describe the reproducibility and the measurements of the outcomes adequately [33]. Additionally, the same study did not present the numerical mean values of the fit or misfit of dentures [33], and only a qualitative summary of the outcomes was described. A pre-determined sample size was used in only two studies [31,32]. Randomization was employed in only one study [33], but the same study did not describe the randomization process and the personnel involved in its implementation. The investigators and the technicians were blinded in only study [31]. Seven studies described their limitations in the discussion section [28,30,32,34,35,36,38]. On the other hand, two studies did not highlight any limitations of the experiments [33,37], and in two studies, it was not clear if the limitations had been described [29,31]. Three studies did not provide any funding information [29,30,32], and none of the studies provided access to the protocol of the study [28,29,30,31,32,33,34,35,36,37,38] (Table 3).

## 4. Discussion

CAD-CAM prostheses provide a significant advantage over conventional cast prostheses in terms of the number of patient visits, appointment duration, and accuracy [21]. Additionally, with the application of intraoral scanning and CAD-CAM, there is no need for impression taking and study or cast model construction, making cross infection easier. The aim of this study was to critically appraise and summarize the current evidence comparing the fit of implant-supported Y-TZP CAD-CAM frameworks to that of other metal and non-metal implant frameworks. The majority of the studies included in this review concluded that implant-supported Y-TZP CAD-CAM frameworks have a better or comparable fit to that of cast and CAD-CAM frameworks constructed from Ti, Co-Cr, resin, and PEEK [28,29,31,35,36,37].

The overall outcome of this systematic review suggests an acceptable fit accuracy of Y-TZP CAD-CAM frameworks, but this should be interpreted with caution due to the heterogeneity in the methodology and outcomes of the studies. Several different CAD-CAM systems were used to construct the frameworks [28,29,30,31,32,33,34,35,36,37,38], making the standardization and comparison of the results difficult. In eight studies, conventional CAD-CAM was used to fabricate frameworks; however, in three studies, copy-milling was employed [28,29,38]. As opposed to conventional CAD-CAM, copy-milling involves the digital scanning of a manually constructed wax or resin pattern of the prostheses. Dimensional changes in the constructed pattern may contribute to discrepancies in the misfit of prostheses constructed with this method. However, to date, no comparative studies have been conducted to assess the misfit of copy-milled Y-TZP frameworks to that of CAD-CAM frameworks. Furthermore, the types of implant abutments used to support the CAD-CAM Y-TZP frameworks [28,29,30,31,32,33,34,35,36,37,38] differed in the reviewed studies, which makes it difficult to prescribe guidelines for constructing CAD-CAM frameworks with an optimal fit or minimal misfit. Another limitation of the studies was that all of them were in vitro laboratory studies [28,29,30,31,32,33,34,35,36,37,38]. Indeed, it is difficult to measure the misfit of prostheses in vivo [44] because there are several factors that affect not only the misfit of implant-supported prostheses but also the overall lifespan of the prostheses. These factors included masticatory forces, parafunctional habits, the age of the patient, systemic health, and the osseointegration of dental implants [45,46,47,48]. Hence, future studies should attempt to simulate the effects of these factors on the misfit of Y-TZP CAD-CAM frameworks.

The differences among the methods used for the assessment of the misfit make it difficult to reach a definite conclusion regarding the misfit of Y-TZP CAD-CAM frameworks to other materials. The ‘one-screw’ test involves the placement of a single screw at the terminal implant abutment, and the opposing abutment is evaluated for movement radiographically or clinically. This test was used in four studies in this review [31,35,36,38]; however, its major limitation is its primary reliance on manual measurements with the naked eye, making the assessments unreliable in many cases. Indeed, this inconsistency is reflected by the results of the four studies that have compared the misfit of Y-TZP CAD-CAM to that of Ti CAD-CAM: in three studies, Y-TZP exhibited either a lower or comparable misfit [31,34,36], and in one study, Ti frameworks possessed a lower misfit [38]. Only two studies made use of CT scanning or virtual scanning to assess the misfit [34,36]. Indeed, the relatively large range of the misfit of the Y-TZP CAD-CAM frameworks (3.7 µm to 103.71 µm) is most likely due to the non-standardization of misfit assessments, so future studies should focus on reproducible and standardized techniques to compare the misfit of frameworks. Nevertheless, due to variations in the fabrication techniques, material phase, and equipment type used, attaining ideal standardization among the Y-TZP misfit studies may not be pragmatic. It is also important to note that CAD-CAM Y-TZP crowns have an approximate success rate of 70% after 24 months, and the most likely reason for this is fatigue-failure [49]. Therefore, more studies focusing on the reasons for CAD-CAM framework misfit and the resultant failures should be conducted. Nevertheless, a recent retrospective clinical study on implant-supported CAD-CAM Y-TZP denture frameworks provided to 50 patients found no long-term failures after 2 years, which makes the long-term viability of Y-TZP CAD-CAM frameworks promising [50]. Nevertheless, for the adequate functionality and survival of implant-supported prostheses, optimal oral hygiene is vital, and patients should be educated about this during and after treatment [51].

In addition to the above concerns, there were multiple sources of bias found in the studies. A pre-determined sample size was used in only two studies [31,32], and the sample sizes in the remaining studies may have not been sufficient to produce reliable results. Furthermore, no study mentioned any attempt in blinding the investigators or technicians during the experiments. Although it is difficult to blind the investigators from the materials due to their difference in appearance, it may be possible to blind the experimental groups corresponding to the measurements of the misfit assessments in future studies. In the majority of the studies, randomization was not attempted, which may have contributed to selection bias within the studies. A major limitation of this systematic review itself was that it was not possible to conduct a meta-analysis because of the heterogeneity of the studies included. Thus, it was not possible to deduce an overall misfit effect of the results. Therefore, to achieve a certain level of standardization among the misfit evaluation investigations, further studies should incorporate blinding, randomization, similar misfit evaluation methods, and analyzed sample sizes.

In addition to CAD-CAM Zirconia frameworks, the 3D printing of such denture frameworks may provide an additional advantage of additive manufacturing leading to the reduced wastage of material and reduced costs [52]. Nevertheless, a lack of clinical trials or other prospective studies to assess the misfit of the Y-TZP CAD-CAM means that, to date, it is difficult to ascertain whether the misfit of these frameworks is lower or comparable to other types of frameworks. Consequently, large-scale clinical studies and standardized in vitro studies with minimal bias are necessary to make a more definite conclusion.

## 5. Conclusions

Within the limitations of this review and the included studies, it may be concluded that Y-TZP CAD-CAM implant-supported frameworks have a comparable misfit to other CAD-CAM implant-supported frameworks. However, due to the heterogeneity in the methodologies of the included studies, the overall numerical misfit of the frameworks tested in the studies is debatable. Better-designed in vitro and long-term clinical studies are required to reach a more definite conclusion.

## Figures and Tables

**Table 1:** General characteristics and the overall outcomes of the studies included.

No.	Study	Groups (*n* = Number of Frameworks Constructed)		Method of Fabrication	Misfit Assessment	Other Assessed Variables	Overall Outcomes
		Test	Control				
1	Abduo et al. 2012 [28]	Y-TZP CAD-CAM (*n* = 5)	Ti CAD-CAM (*n* = 5)	Copy milling (subtractive)	Optical microscopy; Vertical passive fit	Strain	Vertical misfits for Y-TZP and Ti CAD-CAM groups were comparable. Passive misfit for Y-TZP CAD-CAM was significantly lower than that of Ti CAD-CAM. No significant difference in strain among both groups.
2	Zaghloul & Younis et al. 2013 [29]	Y-TZP CAD-CAM (*n* = 10) Y-TZP Copy Milling (n = 10)	Ni-Cr Cast (*n* = 10)	CAD-CAM Copy milling (subtractive)	Digital microscopy; Vertical marginal fit	Cyclic fatigue	Y-TZP CAD-CAM had the highest marginal misfit. No significant difference between Y-TZP copy milling and N-Cr cast frameworks.
3	de França et al. 2014 [30]	Y-TZP CAD-CAM (*n* = 4)	Co-Cr Cast (*n* = 8) Co-Cr CAD-CAM (*n* = 4)	CAD-CAM (milled/subtractive)	SEM; Vertical fit	None	All CAD-CAM frameworks had comparable misfits. CAD-CAM frameworks had significantly lower misfits than cast frameworks.
4	Katsoulis et al. 2014 [31]	Y-TZP CAD-CAM (*n* = 5)	Co-Cr Cast (*n* = 5) Y-TZP-M CAD-CAM (*n* = 5) Ti CAD/AM (*n* = 6)	CAD-CAM (subtractive/milling) Co-Cr cast	One-screw test, SEM; Vertical passive fit	None	No significant difference was observed for vertical misfit between Y-TZP and Ti CAD-CAM, but both were significantly better than Co-Cr.
5	de Araújo et al. 2015 [32]	Group 1: Y-TZP CAD-CAM (*n* = 4)	Co-Cr cast (*n* = 4) Group 2: Co-Cr CAD-CAM (*n* = 4)	CAD-CAM, Cast (milled/subtractive)	SEM; Vertical passive fit	None	Co-Cr CAD-CAM had a significantly lower misfit than the Y-TZP CAD-CAM and Co-Cr Cast specimens. Y-TZP CAD-CAM had a better fit than the cast frameworks.
6	Ghodsi et al. 2018 [33]	Y-TZP CAD-CAM	PEEK CAD-CAM RC CAD-CAM	CAD-CAM (milled/subtractive)	Replica technique; Internal adaptation	Retention force	Y-TZP CAD-CAM had a significantly lower misfit than PEEK and RC. No difference between PEEK and RC misfits.
7	Yilmaz et al. 2018 [34]	Y-TZP CAD-CAM Before and after veneering	Ti CAD-CAM Before and after veneering	CAD-CAM (milled/subtractive)	3D fit (virtual assessment)	None	Y-TZP and Ti CAD-CAM frameworks before and after veneering were comparable. Significant effect of porcelain veneering on Y-TZP frameworks.
8	Yilmaz et al. 2018 [35]	Y-TZP CAD-CAM	HDP CAD-CAM Ti CAD-CAM	CAD-CAM (milled/subtractive)	Marginal misfit; One-screw test	None	HDP had a significantly lower misfit than the Y-TZP and Ti CAD-CAM specimens. No difference between Y-TZP and Ti misfits.
9	Al-Meraikhi et al. 2018 [36]	Y-TZP CAD-CAM (*n* = 5)	Ti CAD-CAM (*n* = 5)	CAD-CAM (milled/subtractive)	Marginal misfit; One-screw test; CT scanning; Color mapping	None	No significant difference between the fits of the Y-TZP and Ti frameworks was observed.
10	da Cunha Fontoura et al. 2018 [37]	Y-TZP CAD-CAM (*n* = 5)	Ti CAD-CAM (*n* = 5)	CAD-CAM (milled/subtractrive)	Vertical misfit; SEM	Torque	No significant difference between the misfits of the Y-TZP and Ti frameworks.
11	Del Rio Silva et al. 2020 [38]	Y-TZP Copy-Milling (*n* = 5)	Ti CAD-CAM (*n* = 5) Co-Cr Cast (*n* = 5)	Co-Cr cast (milled/subtractive)	Marginal fit; One screw test	Stress, loosening torque	Ti had a lower misfit than Y-TZP. Ti and Y-TZP both had lower misfits than Co-Cr. Veneering improved the fit in all groups.

CNC, computer numerical-controlled milling; CAD-CAM, computer-aided design/computer-aided manufacture; HDP, high-density polymer; Ti, titanium; Y-TZP, zirconia; Y-TZP-M, mechanically scanned Zirconia CAD-CAM; Y-TZP-L, laser-scanned zirconia CAD/CM; Ti-L, laser-scanned titanium CAD-CAM; Co-Cr, cobalt-chromium; SEM, scanning electron microscopy; LMC, left maxillary canine; RMC, right maxillary canine; RMFM, right maxillary first molar; PEEK, polyetheretherketone.

**Table 2:** Implant-related characteristics and misfit values in the included studies.

No.	Author	Implant/Abutment System	Implant Dimensions/Location	Implant-Supported Restoration	Fabrication System	Misfit (µm)
1	Abduo et al. 2012 [28]	Mk III TiUnite; Nobel Biocare AB; External hex.	Length: 11.5 mm; diameter: 4.0 mm. First Premolar and second molar on each side	All-on-four full arch fixed denture	Forte, Nobel Biocare, AB (CAD); Fabrication by CAD manufacturer.	Vertical misfit: Y-TZP CAD-CAM: 3.7 µm Ti CAD-CAM: 3.6 µm Passive misfit: Y-TZP CAD-CAM: 5.5 µm Ti: 13.6 µm
2	Zaghloul & Younis et al. 2013 [29]	Friatiz, Dentsply	Length: 11 mm,; diameter: 4–5 mm Second premolar and second molar	Three-unit FPD	Cerec 3 CAD-CAM (Y-TZP); Y-TZP Copy Milling; Ni-Cr Conventional casting	Y-TZP CAD-CAM: 84.58 ± 3.767 µm Y-TZP copy milling: 50.33 ± 3.415 µm Ni-Cr cast: 42.27 ± 3.766 µm
3	de França et al. 2014 [30]	Tapered RP; Nobel Biocare; Internal hex	Titamax Cortical Ti; Neodent Diameter: 4.1 mm; length: 9 mm. Second premolar and second molar	Three-unit FPD	Not specified	Y-TZP CAD-CAM: 5.9 ± 3.6 µm Co-Cr CAD-CAM: 1.2 ± 2.2 µm Co-Cr Cast: Castable abutment: 12.9 ± 11.0 µm Machined abutment: 11.8 ± 9.8 µm
4	Katsoulis et al. 2014 [31]	Replace Select^TM^ Tapered RP; Nobel Biocare	Diameter: 4.3 mm. RMSPM, RMC, RMCI, LMCI, LMC, LMSPM	Ten-unit fixed denture on six implants	CAD: Nobel Biocare (Nobel Procera^TM^); Nobel Biocare CAM: Nobel Procera Production Facility; Nobel Biocare	Y-TZP-L: Median 14 µm 95% CI: 10–26 µm Y-TZP-M: Median 18 µm 95% CI: 12–27 µm Ti-L: Median 15 µm 95% CI: 6–18 µm Co-Cr Cast: Median 236 µm 95% CI: 181–301 µm
5	de Araújo et al. 2015 [32]	Titamax Cortical Ti; Neodent	Diameter: 3.75 mm; length: 9 mm. Three individual implants (second premolar, first molar, second molar)	Three-unit FPD	Not specified	Y-TZP CAD-CAM: 103.81 ± 43.15 µm Co-Cr CAD-CAM: 48.76 ± 13:45 µm Co-Cr Cast: 187.55 ± 103.63 µm
6	Ghodsi et al. 2018 [33]	Not specified	Not described	12 implants (denture details not stated)	CAD: 3Shape; CAM: 3Shape D810 CAD	Y-TZP CAD-CAM: 74.80 µm PEEK CAD-CAM: 181.39 µm RC: 174.89 µm
7	Yilmaz et al. 2018 [34]	Nobel Biocare Active RP	Length: 13 mm; diameter: 4.3 mm. Two straight in the anterior and two distally tilted internal-hexagon dental implants; canine and molar regions	All-on-four fixed denture	CAD: S600 ARTI; Zirkonzahn CAM: M1 Wet Heavy Metal Milling Unit	Before veneering: Y-TZP CAD-CAM: 89 µm T CAD-CAM µm: 88 After veneering: Y-TZP: 175 Ti: 175
8	Yilmaz et al. 2018 [35]	Nobel Biocare Active RP	Length: 13 mm; diameter: 4.3 mm Perpendicular in RMC and LMC; 30-degree distally inclined in RMFM	All-on-four fixed denture	CAD: Zirkonzahn Software; Zirkonzahn CAM: M1 Wet Heavy Metal Milling Unit	RMC HDP: 60 µm Y-TZP CAD-CAM: 83 µm Ti CAD-CAM: 74 µm LMC Not detectable RMFM HDP: 55 µm Y-TZP CAD-CAM: 74 µm Ti CAD-CAM: 102 µm
9	Al-Meraikhi et al. 2018 [36]	Nobel Bioactive	Implants: 4.3 mm × 13 mm Internal Hex	All-on-four fixed denture. Two implants at canine and two implants at first molar positions	CAD: S600 ARTI Zirkonzahn CAM Milling Unit M1 Heavy; Zirkonzahn	LMC-Ti: 8.2 ± 2.6 µm RMC-Ti: 74 ± 15 µm RMC-Y-TZP: 84.4 ± 12.1 µm RMFM-Ti: 102 ±26.7 µm RMFM-Y-TZP: 93.8 ± 30 µm
10	da Cunha Fontoura et al. 2018 [37]	ITI Straumann	Diameter 4.1; length: Not available. Location: mandibular-2 at central incisors and 2 at canines	All-on-four. First premolar to first premolar	CAD: Zirkozahn Modellier; Zirkozahn CAM: Milling Unit M5 Heavy; Zirkonzahn	Ti CAD-CAM: 6.011 ± 0.750 µm Y-TZP CAD-CAM: 9.055 ± 3.692 µm
11	Del Rio Silva et al. 2020 [38]	Easy Grip Porous EH	Implants: 4.1 mm × 11.5 mm (premolar region), 4.1 mm × 11.5 mm (incisor region), 5 mm × 7 mm (molar region)	Fixed complete denture supported by six implants	Ceramill Map 400+; Amann Girrbach/Ceramill Motion 2; Amann Girrbach (Y-TZP) and CNC D15W; Yenadent (Co-Cr & Ti)	Mean values not provided. Ti CAD-CAM had the highest fit before veneering. No difference in fit after veneering.

CAD, computer-assisted design; CAM: computer-assisted manufacture; Y-TZP, zirconia; Ti, titanium; Co-Cr, cobalt-chromium; Ni-Cr, nickel-chromium; RMSPM, right maxillary second premolar; RMC, right maxillary canine; RMCI, right maxillary central incisor; LMCI, left maxillary central incisor; LMC, left maxillary canine; LMSPM, left maxillary second premolar; PEEK, polyetheretherketone.

**Table 3:** Results of the quality assessment conducted on the studies included in this review.

Assessment Item	Abduo et al. 2012 [28]	Zaghloul & Younis et al. 2013 [29]	de França et al. 2014 [30]	Katsoulis et al. 2014 [32]	de Araújo et al. 2015 [32]	Ghodsi et al. 2018 [33]	Yilmaz et al. 2018 [34]	Yilmaz et al. 2018b [35]	Al-Meraikhi et al. 2018 [36]	Diego et al. 2018 [37]	Silva et al. 2020 [38]
1. Adequate abstract	Yes	Yes	Yes	Yes	Yes	Yes	Yes	Yes	Yes	Yes	Yes
(2a) Introduction (Background)	Yes	Yes	Yes	Yes	Yes	Not clear	Yes	Yes	Yes	Yes	Yes
(2b) Introduction (Objectives)	Yes	Yes	Yes	Yes	Yes	Not clear	Yes	Yes	Yes	Yes	Yes
Methods											
3. Replicable methods	Yes	Yes	Yes	Yes	Yes	Not clear	Yes	Yes	Yes	Yes	Yes
4. Adequate outcomes	Yes	Yes	Yes	Yes	Yes	Not clear	Yes	Yes	Yes	Yes	Yes
5. Pre-determined sample size	No	No	No	Yes	Yes	No	No	No	No	Yes	Yes
6. Allocation of samples											
(a) Randomization	No	No	No	No	No	Yes	No	No	No	No	No
(b) Allocation concealment	No	No	No	No	No	No	No	No	No	No	No
(c) Implementation	No	No	No	No	No	No	No	No	No	No	No
7. Blinding	No	No	No	Yes	No	No	No	No	No	No	No
8. Statistics	Yes	Yes	Yes	Yes	Yes	Yes	Yes	Yes	Yes	Yes	Yes
9. Adequate outcomes & estimation	Yes	Yes	Yes	Yes	Yes	Yes	Yes	Yes	Yes	Yes	Yes
10. Discussion: Limitations	Yes	Not clear	Yes	Not clear	Yes	No	Yes	Yes	Yes	No	Yes
11. Funding	Yes	No	No	Yes	No	Yes	Yes	Yes	Yes	Yes	Yes
12. Accessible protocol	No	No	No	No	No	No	No	No	No	No	No
Overall quality	Medium	Medium	Medium	High	Medium	Low	Medium	Medium	Medium	Medium	High

## Data Availability

The data are available upon request from the author.
